# Efficient supersonic flow simulations using lattice Boltzmann methods based on numerical equilibria

**DOI:** 10.1098/rsta.2019.0559

**Published:** 2020-06-22

**Authors:** Jonas Latt, Christophe Coreixas, Joël Beny, Andrea Parmigiani

**Affiliations:** 1 Department of Computer Science, University of Geneva, 1204 Geneva, Switzerland; 2 FlowKit-Numeca Group Ltd, Route d’Oron 2, 1010 Lausanne, Switzerland

**Keywords:** lattice Boltzmann method, CFD, compressible, supersonic, CPU, GPU

## Abstract

A double-distribution-function based lattice Boltzmann method (DDF-LBM) is proposed for the simulation of polyatomic gases in the supersonic regime. The model relies on a numerical equilibrium that has been extensively used by discrete velocity methods since the late 1990s. Here, it is extended to reproduce an arbitrary number of moments of the Maxwell–Boltzmann distribution. These extensions to the standard 5-constraint (mass, momentum and energy) approach lead to the correct simulation of thermal, compressible flows with only 39 discrete velocities in 3D. The stability of this BGK-LBM is reinforced by relying on Knudsen-number-dependent relaxation times that are computed analytically. Hence, high Reynolds-number, supersonic flows can be simulated in an efficient and elegant manner. While the 1D Riemann problem shows the ability of the proposed approach to handle discontinuities in the zero-viscosity limit, the simulation of the supersonic flow past a NACA0012 aerofoil confirms the excellent behaviour of this model in a low-viscosity and supersonic regime. The flow past a sphere is further simulated to investigate the 3D behaviour of our model in the low-viscosity supersonic regime. The proposed model is shown to be substantially more efficient than the previous 5-moment D3Q343 DDF-LBM for both CPU and GPU architectures. It then opens up a whole new world of compressible flow applications that can be realistically tackled with a purely LB approach.

This article is part of the theme issue ‘Fluid dynamics, soft matter and complex systems: recent results and new methods’.

## 1. Introduction

The lattice Boltzmann method (LBM) is a popular numerical scheme capable of computing solutions to the Boltzmann equation (BE) in a regime of small deviations from the local equilibrium state [1,2]. It offers a pathway to recover the solutions of the incompressible Navier–Stokes or compressible Navier–Stokes–Fourier equations [3–5]. It can therefore be used as an alternative to classical solvers in the field of Computational Fluid Dynamics and has gained traction notably in areas with complex and coupled physics. Examples include multi-phase [6–8] or particulate flows [9], or biomedical applications [10,11]. A review of the method is for example provided in [4,5,12]. In the BE, the statistical behaviour of gas molecules is described by the continuous velocity distribution function *f*(***x***, **ξ**, *t*) that depends on the spatial position ***x***, the molecular velocity **ξ** and time *t* [13]. In the LB scheme, the space of molecular velocities is discretized: the velocity distribution function is replaced by a discrete set of *V* populations *f*_*i*_(***x***, *t*), standing for the statistics of molecules at discrete velocities **ξ**_*i*_ (*i* = 0 · · · *V* − 1). Increasing the number of discrete velocities, and thus populations, typically allows us to extend the physical range of validity of the scheme [14].

Although the LBM describes intrinsically compressible fluids, it has arguably achieved its most striking successes in the realm of incompressible fluid flow [4,5] and has until recently struggled to establish itself as a realistic alternative for the simulation of compressible and/or high Mach number flows [3]. While simulating fluid flow may seem as easy as adding discrete velocities to the LBM scheme, these so-called multi-speed approaches rapidly lead to schemes with a large number of velocities, which translate to an impractically large number of degrees of freedom per mesh cell. In addition to that, these models are usually limited by numerical instabilities [15], except if the numerical scheme is modified [3], or if a more robust collision model is adopted [16–18].

As a popular workaround, the momentum and energy equations can be split and solved separately, as they individually require a substantially smaller set of velocities [19,20]. Further computational efficiency is achieved by solving the energy equation with a traditional finite-difference, finite-volume or finite-element scheme [21,22]. However, these solutions introduce new problems of their own making in the shape of coupling instabilities, that can be reduced through a careful choice of the coupling methodology [23].

Another interesting path has been taken by the community of Discrete Velocity Models (DVMs) to derive kinetic models devoted to the simulation of rarefied gas flows [24–35], even though it is not restricted to this application field [28,36]. A compact review of that topic was proposed by Mieussens in 2014 [37]. As ancestors of LBMs, DVMs also solve a discrete velocity BE (DVBE). They are based on large discretizations of the velocity space, more complex numerical schemes (finite-volume, finite-element, discontinuous Galerkin, etc.), and they rely on discrete equilibrium functions in the form of an exponential that are derived through the maximum entropy principle (MEP [26,27,29,38–40]). In the context of rarefied gas flow, low-order DVMs compute this exponential equilibrium on each cell and at each time step by solving a set of non-linear equations, subject to only five constraints (mass, momentum and energy conservation [29,32,33]). On the contrary, high-order models can incorporate more constraints to increase their validity range [26,27], as proposed by Levermore in his seminal work on the derivation of high-order closure of kinetic theories [39]. Existence, uniqueness and positiveness of solutions to this problem, by a root-finding algorithm like Newton–Raphson, have been extensively discussed by authors of the DVM. Best practices were also provided to accelerate the convergence and increase the robustness of the root-finding algorithm [29,30]. Nevertheless, DVMs are computationally expensive due to the great number of discrete velocities—which usually exceeds a thousand in 3D—to recover the correct shape of populations in out-of-equilibrium regions of the simulation domain, and to reduce as much as possible macroscopic errors related to unconstrained moments (starting from second-order ones for the 5-moment approach).

More recently, the model based on two sets of populations and the 5-constraint based equilibrium [32] has been (re)introduced in the context of compressible entropic LBMs by Frapolli *et al.* [41,42]. Among DDF-LBMs based on the collide-and-stream algorithm, this is to our knowledge one of the most promising propositions in the current literature capable of achieving good numerical stability for non-trivial, transonic and supersonic flows. As for DVMs, this LBM suffers from a large number of macroscopic errors induced by the 5-constraint equilibrium, and which can only be reduced by using large velocity discretizations. Hence, the increased stability provided by this type of equilibrium seems to come at a very high computational cost even in the LB context, requiring typically two populations composed of 343 velocities each to recover the correct macroscopic behaviour in the supersonic regime. As compared to the conventional 19 or 27 velocities needed to simulate weakly compressible and isothermal flows, such an approach rapidly becomes prohibitive and faces severe difficulties to simulate realistic configurations in an acceptable time frame, or using an acceptable amount of hardware resources. This is because grid meshes composed of tens, or even hundreds of millions of points are usually needed to achieve the high accuracy required at an industrial level. Finally, the approach relies on the entropic collision model that requires minimizing the H-functional, on every single grid point and at every time iteration, in order to get more stable simulations. This eventually leads to a further non-negligible overhead.

In this publication, we extend the above compressible LBM by deriving exponential (numerical) discrete equilibria designed to recover an *arbitrary* number of the Maxwellian moments, with particular emphasis on the 13-moment approach. This follows an idea proposed in the context of DVMs by Le Tallec & Perlat in 1997 [26], and which was implemented 20 years later within an LBM collision-streaming scheme in the PhD manuscript of Frapolli [43]. With 13 constraints, the equilibrium distribution matches the moments of the Maxwell–Boltzmann distribution not only for the conserved moments, as does the 5-moment approach, but also for higher-order, non-conserved moments. In this manner, the behaviour of the macroscopic flow variables, which are related to the moments of the particle populations, can be more adequately represented using as few as 39 discrete velocities in 3D, by putting more effort into the equilibrium instead of the lattice. This is explained by the fact that the desired moments of the distribution are strictly enforced with the help of a numerical solver. Hence, they no longer depend on the choice of a sophisticated discrete velocity stencil, or at least, the dependency is highly reduced.

Here, the choice of a D3Q39 velocity discretization is motivated by recent improvements obtained with hybrid LBMs based on the same lattice, and which successfully simulated flows around realistic geometries in the high-subsonic, transonic and supersonic regimes with a wall-clock time that competes with Navier–Stokes–Fourier solvers [22,44,45]. Thanks to this reduced number of discrete velocities, the memory requirements are reduced by an order of magnitude, and the computational cost is diminished correspondingly as compared to the D3Q343 5-constraint method. Nevertheless, the D3Q39 is only one possibility among many available lattices. For example, D3Q19-LBMs are particularly suited for HPC architectures [46], and even in their isothermal form, they are serious alternatives to Navier–Stokes–Fourier solvers in the industrial context [47,48]. Hence, standard lattices are also perfect candidates for a coupling with numerical equilibria.

In contrast to the entropic collision model required by Frapolli *et al.* to obtain stable simulations in the low-viscosity limit [41], we rely on BGK operators [49] that are stabilized by identifying areas of the simulation domain where the departure from equilibrium is high. In doing so, relaxation times can be adjusted to damp high-order modes, which efficiently leads to stable and accurate simulation of supersonic flows with discontinuities. The latter points confirm that a proper control of under-resolved phenomena through subgrid-scale models supplemented with shock-capturing techniques is a viable and efficient alternative to the computationally intensive entropic collision. Consequently, the proposed model combines the elegance of a fully LB model for compressible flows with a level of robustness and efficiency.

The rest of the paper reads as follows. First, the derivation of discrete equilibrium based on the maximum entropy principle is recalled in the context of fluid mechanics (§2). It is followed by the description of the numerical discretization, as well as the kinetic sensor. Then, a parametric study, involving several lattices and sets of constraints, is proposed in §3. It is based on the evaluation of macroscopic errors related to each combination (lattice and number of constraints), and it aims at finding the best model in terms of accuracy, stability and efficiency in the context of supersonic flow simulations. Successive numerical tests shown in this article (1D Riemann problem, and supersonic flow past a 2D aerofoil and a 3D sphere) confirm that the model exhibits both a good stability and accuracy with this reduced velocity set (§4). The computational expense of the method is measured on both CPU and GPU hardwares in §5. Conclusions and perspectives are eventually provided in §6.

For the sake of completeness, details regarding the derivation of all numerical equilibria considered in this work are gathered in appendices A and B. Eventually, shifted lattices are discussed in the context of numerical equilibria in appendix C.

## 2. Compressible LBMs based on numerical equilibria

### (a) From macroscopic equations to moments of the Maxwellian

The Maxwell–Boltzmann distribution

2.1 $$f^{\text{eq}}=\frac{\rho }{{\left(2\pi T\right)}^{D/2}}\exp\left[-\frac{{\left(\xi -u\right)}^{2}}{2T}\right],$$

describes an equilibrium state and yields an exact solution to the BE in *D*-dimension. The macroscopic density *ρ*, velocity ***u*** and temperature *T* are equal to or directly linked with velocity moments of the probability distribution function. It is easily verified that for the continuum Maxwell–Boltzmann distribution, density, velocity and total energy are conserved quantities since the corresponding moments of the distribution function *f* and of the equilibrium state *f*^eq^ are equal.

In the discrete LB method, the equilibrium populations $f_{i}^{\text{eq}}$ are a mere approximation of the Maxwell–Boltzmann distribution, but most LB models guarantee the correct definition of conserved macroscopic quantities after the velocity space discretization. To achieve this goal, the most common methodology is to rely on Gauss–Hermite quadrature [14]. In this case, the match between moments and macroscopic variable is nothing other than a statement of orthogonality between Hermite polynomials. However, the number of discrete velocities required to express appropriate orthogonality relations and include effects of energy conservation can be prohibitively large (at least 100 velocities in 3D). As an alternative originating from DVMs [26,27,29], other authors proposed to manually enforce conservation laws by constantly recomputing the equilibrium by a root-finding procedure subject to appropriate constraints [41]. It is also interesting to note that lattice gas cellular automota (LGCA), based on the same principle, were also proposed by Kornreich & Scalo for the simulation of supersonic flows in 1993 [50]. Nevertheless, these LGCA were rapidly forgotten whereas DVMs based on numerical equilibria remain an active research field, especially in the context of rarefied gas flow dynamics [37].

Beyond conserved quantities, it is however necessary for the moments of the discrete equilibrium to yield the same value as the corresponding moments of the Maxwell–Boltzmann distribution in order to recover the desired physical behaviour. We now provide a short reasoning for this argument, and compute the order of the moments that need to be recovered exactly to retrieve the Navier-Stokes-Fourier (NSF) level of physics. The compressible NSF equations read

2.2 $$\begin{array}{rl} & \partial_{t}\rho +\partial_{\chi }(\rho u_{\chi })=0,\\ & \partial_{t}(\rho u_{\alpha })+\partial_{\beta }(\rho u_{\alpha }u_{\beta }+p\delta_{\alpha \beta })=\partial_{\beta }(\Pi_{\alpha \beta })\\ \text{and}\;\; & \partial_{t}(\rho E)+\partial_{\alpha }((\rho E+p)u_{\alpha })=\partial_{\alpha }(\Phi_{\alpha }), \end{array}\}$$

where the index repetition implies the Einstein summation rule. The viscous stress tensor Π_*αβ*_ is defined as

$$\Pi_{\alpha \beta }=\mu (S_{\alpha \beta }-\frac{2}{D}\partial_{\chi }u_{\chi }\delta_{\alpha \beta })+\mu_{b}\partial_{\chi }u_{\chi }\delta_{\alpha \beta },$$

with *S*_*αβ*_ = ∂_*α*_*u*_*β*_ + ∂_*β*_*u*_*α*_, *μ* and *μ*_*b*_ = (2/*D* − 1/*C*_*v*_)*μ* being the dynamic and bulk viscosity, respectively. *D* is the number of physical dimensions, *C*_*v*_ = 1/(*γ*_*r*_ − 1) is the heat capacity at constant volume, *γ*_*r*_ is the heat capacity ratio, and *E* is the total energy. Dissipative effects in the total energy equation are gathered in the term

$$\Phi_{\alpha }=-\lambda \partial_{\alpha }T+\Pi_{\alpha \beta }u_{\beta },$$

which accounts for both the Fourier heat flux (*λ* is the heat conductivity), and the viscous heat dissipation. In the case of a calorically perfect gas, this system is closed by the following equation of state: $E=\frac{1}{2}u^{2}+C_{v}T$.

The moments of the Maxwell–Boltzmann distribution can be written as

2.3 $$M_{pqr}^{\text{MB}}=\int \xi_{x}^{p}\xi_{y}^{q}\xi_{z}^{r}f^{\text{eq}}\;\text{d}\xi .$$

For comparison with macroscopic equations, it is more convenient to write them in the following compact form:

2.4 $$\begin{array}{rl} & M_{0}^{\text{MB}}=\rho ,\\ & M_{1,\alpha }^{\text{MB}}=\rho u_{\alpha },\\ & M_{2,\alpha \beta }^{\text{MB}}=\rho u_{\alpha }u_{\beta }+\rho T\delta_{\alpha \beta },\\ & M_{3,\alpha \beta \gamma }^{\text{MB}}=\rho u_{\alpha }u_{\beta }u_{\gamma }+\rho T{\left[u_{\alpha }\delta_{\beta \gamma }\right]}_{\text{cyc}}\\ \text{and}\;\; & M_{4,\alpha \beta \gamma \chi }^{\text{MB}}=\rho u_{\alpha }u_{\beta }u_{\gamma }u_{\chi }+\rho T{\left[u_{\alpha }u_{\beta }\delta_{\gamma \chi }\right]}_{\text{cyc}}+\rho T^{2}{\left[\delta_{\alpha \beta }\delta_{\gamma \chi }\right]}_{\text{cyc}}, \end{array}\}$$

where *α*, *β*, *γ*, *χ* represent space coordinates (*x*, *y* or *z*). The subscript cyc labels a cyclic permutation without repetition. As an example,

$$T^{2}{\left[\delta_{\alpha \beta }\delta_{\gamma \chi }\right]}_{\text{cyc}}=T^{2}(\delta_{\alpha \beta }\delta_{\gamma \chi }+\delta_{\alpha \gamma }\delta_{\beta \chi }+\delta_{\alpha \chi }\delta_{\beta \gamma }).$$

Now, the NSF equations (2.2) are rewritten in terms of equilibrium moments as follows:

2.5 $$\begin{array}{rl} & \partial_{t}(M_{0}^{\text{MB}})+\partial_{\beta }(M_{1,\beta }^{\text{MB}})=0, \end{array}$$

2.6 $$\begin{array}{rl} & \partial_{t}(M_{1,\alpha }^{\text{MB}})+\partial_{\beta }(M_{2\alpha \beta }^{\text{MB}})\propto \partial_{t}(M_{2\alpha \beta }^{\text{MB}})+\partial_{\chi }(M_{3,\alpha \beta \chi }^{\text{MB}}), \end{array}$$

2.7 $$\begin{array}{rl} \text{and}\;\; & \partial_{t}(M_{2\alpha \alpha }^{\text{MB}})+\partial_{\beta }(M_{3,\alpha \alpha \beta }^{\text{MB}})\propto \partial_{t}(M_{3,\alpha \chi \chi }^{\text{MB}})+\partial_{\beta }(M_{4,\alpha \beta \chi \chi }^{\text{MB}}). \end{array}$$

Here, the diffusive r.h.s. terms have been related to equilibrium moments through the Chapman–Enskog expansion, where the time derivatives are usually replaced using Euler-level equations for $M_{2,\alpha \beta }^{\text{MB}}$ and $M_{3,\alpha \chi \chi }^{\text{MB}}$. From this, it is clear that moments of *f*^eq^ up to *n* = 4 are necessary to recover NSF equations (2.2) in the continuum limit [14,51].

It is finally worth noting that the above methodology allows us to recover the behaviour of monatomic gases (*C*_*v*_ = *D*/2) with a Prandtl number fixed to Pr = 1. The former limitation can be overcome through the use of a second population [52], while the latter problem is usually corrected by changing the collision model [53,54].

### (b) From discrete velocity models to lattice Boltzmann method

A common, deterministic and efficient way to solve the (force-free) BGK-BE

2.8 $$\partial_{t}f+\partial_{\alpha }(\xi_{\alpha })=-\frac{1}{\tau }(f-f^{\text{eq}}),$$

is to rely on a discretization of the velocity space, which leads to the discrete velocity Boltzmann equation (DVBE) based on the BGK operator [37]

2.9 $$\forall i\in \{1,\ldots ,V\},\;\partial_{t}f_{i}+\partial_{\alpha }(\xi_{i,\alpha })=-\frac{1}{\tau }(f_{i}-f_{i}^{\text{eq}}).$$

If the above velocity discretization is done in such a way that the matching conditions between discrete and continuous moments

2.10 $$\sum_{i}\;f_{i}^{\text{eq}}\xi_{x}^{p}\xi_{y}^{q}\xi_{z}^{r}=M_{pqr}^{\text{MB}}$$

are satisfied up to the order *p* + *q* + *r* = *n*, then the macroscopic behaviour of interest is automatically satisfied up to the same order *n* [2,14]. In fact, there are several ways to enforce them. The most popular ones rely on the maximum entropy principle (MEP), the moment-matching approach or the (Gauss–Hermite) quadrature-based methodology. For the latter two approaches, strict conditions must be satisfied by the velocity discretization in order to obtain an *analytical* formulation of the discrete equilibrium $f_{i}^{\text{eq}}$ [3–5]. Interestingly, in the context of the MEP, it is possible to obtain a general formulation of the discrete equilibrium that *does not necessarily depend on the velocity discretization considered*. This particular form of the equilibrium is obtained considering the following *H*-function [55]

2.11 $$H=\sum_{i}\;f_{i}\left[\ln\left(\frac{f_{i}}{a}\right)\right],$$

alongside the set of constraints

2.12 $$G_{pqr}=\sum_{i}\;f_{i}^{\text{eq}}\xi_{x}^{p}\xi_{y}^{q}\xi_{z}^{r}-M_{pqr}^{\text{MB}}=0,$$

and reads as

2.13 $$f_{i}^{\text{eq}}=\rho \exp\left[-\left(1+\sum_{p,q,r}\lambda_{M_{pqr}^{\text{MB}}}\xi_{x}^{p}\xi_{y}^{q}\xi_{z}^{r}\right)\right],$$

where $\lambda_{M_{pqr}^{\text{MB}}}$ are the Lagrange multipliers corresponding to the constraints (2.12), and *a* = *ρ* is a possible choice in the context of fluid flow simulations—even though the latter choice is not mandatory, e.g. DVMs rely on *a* = 1 for the simulation of rarefied gas flow [29]. By enforcing the conservation of mass, momentum and energy via

2.14 $$G_{\rho }=\sum_{i}f_{i}-\rho =0,\;G_{\rho u_{\alpha }}=\sum_{i}f_{i}\xi_{i,\alpha }-\rho u_{\alpha }=0,\;G_{\rho E}=\sum_{i}f_{i}\xi_{i}^{2}-2\rho E=0,$$

one ends up, in the 3D case, with the exponential equilibrium of the 5-moment approach

2.15 $$f_{i}^{\text{eq}}=\rho \exp[-(1+\lambda_{\rho }+\lambda_{\rho u_{\alpha }}\xi_{i,\alpha }+\lambda_{\rho E}\xi_{i}^{2})],$$

where *λ*_*ρ*_, $\lambda_{\rho u_{\alpha }}$, *λ*_*ρE*_ are the five corresponding Lagrange multipliers. The interested reader may refer to appendix A for the derivation of both equilibria (2.13) and (2.15), as well as a brief discussion on the scaling factor *a*.

The above minimization problem admits exact solutions for some velocity discretizations (D1Q3, D2Q9, D3Q27) [56,57]. Nevertheless, finding an exact solution to this optimization problem is not guaranteed in the more general case where a number *M* of constraints must be satisfied. In order to obtain exact solutions, a popular workaround is to expand the discrete equilibrium with respect to Lagrange multipliers (see [58–60] and therein references). Yet, this methodology is lattice-dependent and consequently requires us to derive the discrete equilibrium for every single lattice considered. As a very powerful solution to this issue, researchers belonging to the community of DVMs proposed to obtain *numerical* discrete equilibria in such a way that the resulting algorithm would not explicitly depend on the number of discrete velocities [26–37]. This is achieved by finding an approximate *numerical* solution to the optimization problem through multivariate root-finding algorithms.

One can refer to Le Tallec & Perlat [26], and Mieussens [29], for implementations of the monatomic 14- and 5-constraint based DVMs, published in 1997 and 2000, respectively. Their polyatomic extensions were proposed by Andries *et al.* [61] and Dubroca *et al.* [32] in the early 2000s. Both DVMs rely on a second population *g*_*i*_, which accounts for additional internal degrees of freedom *K* (rotational and vibrational) through

2.16 $$g_{i}^{\text{eq}}=(2C_{v}-D)T\;f_{i}^{\text{eq}},$$

with the heat capacity at constant volume *C*_*v*_ = (*D* + *K*)/2 and the heat capacity ratio *γ*_*r*_ = 1 + 2/(*D* + *K*). Several ways of obtaining the correct Prandtl number with DVMs were also proposed [26,33,61].

For DVMs, the velocity discretization is not necessarily linked to the numerical discretization in both space and time (off-grid lattice). Since these approaches also rely on the splitting between the convective and collision parts of the DVBE, populations *f*_*i*_ might end up in between grid nodes after the convection step. To avoid such an issue, finite-volume approaches are usually considered to ensure the conservative properties of the numerical discretization when on-grid lattices are not adopted. Interestingly, when the stream-and-collide algorithm is considered for the numerical discretization of DVMs, one ends up with the standard definition of LBMs based on either on- or off-grid lattices. This strong relationship between DVMs and LBMs recently led Aristov *et al.* [62–64] to couple both approaches in order to achieve accurate simulations of flows from the continuum to the rarefied regime, as originally proposed by Succi [65].

In the context of on-grid lattice based LBMs, Frapolli *et al.* proposed in 2015 a stream-and-collide version of the polyatomic DVM [32], and further coupled it to the entropic collision model in order to compensate for the issues encountered by the BGK-LBM in the low-viscosity regime [41]. In their work, the velocity discretization was chosen in order to minimize as much as possible the errors with respect to unconstrained macroscopic moments. The latter study led to the D3Q343 velocity discretization which seemed to be a good trade-off between accuracy and efficiency from the authors’ point of view. Nevertheless, such a lattice cannot be used in an industrial context due to its tremendous requirements in terms of memory storage and simulation time, as compared to Navier–Stokes–Fourier solvers.

### (c) High-order numerical equilibria

One way to circumvent this issue is to put more effort into the computation of the discrete equilibrium, instead of simply increasing the size of the lattice. To do so, one needs to drop the 5-moment formulation (2.15) and adopt the more general equilibrium (2.13). The next step consists of choosing the number of constraints that should be satisfied. Such constraints can be found in the works dedicated to high-order closures based on the MEP for the description of rarefied gas dynamics. The latter includes works on both DVMs [39] and moment methods [55,66]. For example, the seminal paper by Levermore [39] introduces sets of admissible moments whose maximal order is either two or four:

−5-moment:
  2.17 $$(1,\;\xi_{i\alpha },\;\xi_{i\chi }\xi_{i\chi }),$$
−10-moment:
  2.18 $$(1,\;\xi_{i\alpha },\;\xi_{i\alpha }\xi_{i\beta }),$$
−14-moment:
  2.19 $$(1,\;\xi_{i\alpha },\;\xi_{i\alpha }\xi_{i\beta },\;\xi_{i\chi }\xi_{i\chi }\xi_{i\alpha },\;\xi_{i\chi }\xi_{i\chi }\xi_{i\eta }\xi_{i\eta }),$$
−21-moment:
  2.20 $$\left(1,\;\xi_{i\alpha },\;\xi_{i\alpha }\xi_{i\beta },\;\xi_{i\alpha }\xi_{i\beta }\xi_{i\gamma },\;\xi_{i\chi }\xi_{i\chi }\xi_{i\eta }\xi_{i\eta }\right)$$
−26-moment:
  2.21 $$(1,\;\xi_{i\alpha },\;\xi_{i\alpha }\xi_{i\beta },\;\xi_{i\alpha }\xi_{i\beta }\xi_{i\gamma },\;\xi_{i\chi }\xi_{i\chi }\xi_{i\alpha }\xi_{i\beta }),$$
−35-moment:
  2.22 $$(1,\;\xi_{i\alpha },\;\xi_{i\alpha }\xi_{i\beta },\;\xi_{i\alpha }\xi_{i\beta }\xi_{i\gamma },\;\xi_{i\alpha }\xi_{i\beta }\xi_{i\gamma }\xi_{i\chi }).$$

In fact, the above sets enclose all *linear* combinations of moments belonging to each set, i.e. their raw, Hermite, central and central Hermite version—*a fortiori*, it does not extend to *non-linear* combinations of moments such as cumulants. In addition to Levermore’s sets of moments, restrictions to third-order moments (and their *linear* combinations) were proposed in the context of moment approaches:

−13-moment:
  2.23 $$(1,\;\xi_{i\alpha },\;\xi_{i\alpha }\xi_{i\beta },\;\xi_{i\chi }\xi_{i\chi }\xi_{i\alpha }),$$
−20-moment:
  2.24 $$(1,\;\xi_{i\alpha },\;\xi_{i\alpha }\xi_{i\beta },\;\xi_{i\alpha }\xi_{i\beta }\xi_{i\gamma }),$$

where Grad’s 13 moments can be obtained from the set (2.23) by considering second- and third-order moments based on peculiar discrete velocities ${\bar{\xi }}_{i\alpha }=\xi_{i\alpha }-u_{\alpha }$ [66–68].

In the context of LBMs based on the numerical equilibrium (2.13), each set of moments (2.17)–(2.24) leads to a different macroscopic behaviour. Roughly speaking, the asymptotic limit recovered by LBMs based on the above sets of moments can be summarized as

2.25 $$\begin{array}{rl} & \partial_{t}\rho +\partial_{\chi }(\rho u_{\chi })=0,\\ & \partial_{t}(\rho u_{\alpha })+\partial_{\beta }(\rho u_{\alpha }u_{\beta }+p\delta_{\alpha \beta })+\Delta_{2}=\partial_{\beta }(\Pi_{\alpha \beta })+\Delta_{3}\\ \text{and}\;\; & \partial_{t}(\rho E)+\partial_{\alpha }((\rho E+p)u_{\alpha })+\Delta_{3}^{\text{tr}}=\partial_{\alpha }(\Phi_{\alpha })+\Delta_{4}^{\text{tr}}, \end{array}\}$$

where Δ_*n*_ and $\Delta_{n}^{\text{tr}}$ are errors, respectively, related to equilibrium moments of order *n* and their trace, that would emerge if the corresponding constraints are not accounted for in the computation of the exponential equilibrium (2.13). Hence, the set of 13 moments (2.23) is the minimal configuration allowing us to *exactly* recover the Euler equations ($\Delta_{2}=\Delta_{3}^{\text{tr}}=0$). Regarding the Navier–Stokes–Fourier equations, one needs to further account for constraints related to $M_{\alpha \beta \gamma }^{\text{MB}}$ and $M_{\alpha \beta \chi \chi }^{\mathrm{MB}}$ in order to *exactly* recover this asymptotic limit. The 26 constraints based on equation (2.21) lead to the minimal configuration that satisfies these conditions.

It is important to understand that the above reasoning is only valid if one wants to *exactly* recover the macroscopic behaviour of interest. In practice, it is not necessary to satisfy all the required constraints on the Maxwellian moments to obtain accurate numerical solvers. As an example, the 5-moment approach is the cornerstone of most DVMs, and it allows them to accurately simulate rarefied gas flow dynamics [37]. Nevertheless, this can only be achieved by drastically increasing the size of the velocity discretization. The same approach was followed by Frapolli *et al.* [41,42], and led the authors to choose the D3Q343 lattice.

In addition, one must bear in mind that for the present approach, the numerical equilibrium (2.13) must be computed at each grid node and for every time step. Hence, increasing the number of constraints will increase the size of the optimization problem accordingly, and *a fortiori*, impact the simulation time of the present approach. Furthermore, the root-finding algorithm used for the latter computation will encounter more difficulties to converge towards an adequate solution if the number of constraints that needs to be enforced is high.

Consequently, an in-depth investigation is required in order to find a good trade-off, in terms of both lattice size and number of constraints, to accurately and efficiently simulate supersonic flows in a stable manner.

### (d) Numerical method

Similarly to DVMs, the present approach relies on the computation of a numerical equilibrium which results from an optimization problem under *M* constraints. The latter constraints are used to enforce the correct form of the discrete equilibrium through its moments. The different steps required for the computation of the numerical equilibrium are [29]

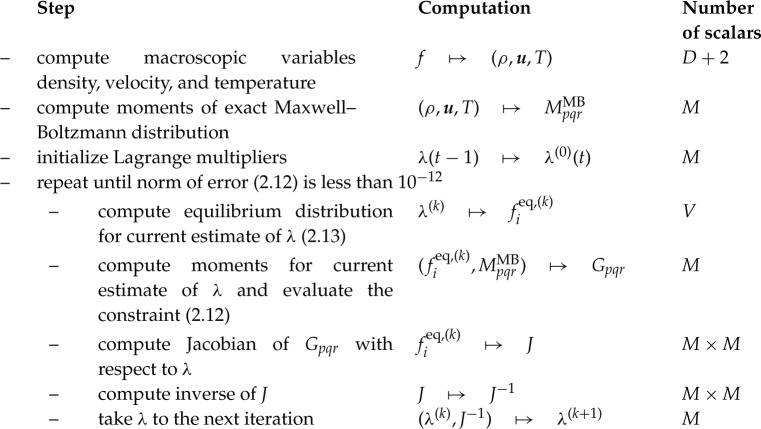

with *M* and *V* being the number of constraints (or moments) and discrete velocities, respectively. In this work, only 3D models are considered hence *D* = 3. The convergence criterion is of utmost importance as it ensures that constraints are correctly enforced with an accuracy close to machine precision. By taking values greater than 10^−12^, one would obviously end up with a faster convergence of the root-finding algorithm, but it would be at the cost of conservation issues.

Hereafter, contrarily to DVMs, the collide-and-stream algorithm is adopted to obtain an efficient and conservative numerical discretization of the DVBE (2.9). Only velocity discretizations that include up to three layers of discrete velocities (i.e. max _*i*_*ξ*_*i*,*α*_ ≤ 3 for *α* = *x*, *y* and *z*) are considered to not drastically deteriorate the parallel efficiency of the resulting LBM: D3Q15, D3Q19, D3Q27, D3Q39, D3Q125 and D3Q343 (their detailed structure is summarized in table 1). For the sake of compactness, the notation ‘D3’ is dropped in the rest of the paper.

**Table 1.** Description of lattice structures of interest. Velocity groups included in each lattice compose the right part of the table, and *p* is the number of discrete velocities composing each group. Here, the cyclic permutation is implied, which means for example that (1, 0, 0) stands for all six possibilities ( ± 1, 0, 0), (0, ± 1, 0) and (0, 0, ± 1). The reference temperatures *T*_0_ corresponding to each lattice are (from left to right): 1/3, 1/3, 1/3, 2/3, $1\pm \sqrt{2/5}$ and 0.697953322019683. Data are compiled from the works by Karlin & Asinari [69] and Shan [70].

group	$\xi_{i}$	*p*	Q15	Q19	Q27	Q39	Q125ZOT	Q343
1	(0, 0, 0)	1	X	X	X	X	X	X
2	(1, 0, 0)	6	X	X	X	X	X	X
3	(1, 1, 0)	12		X	X		X	X
4	(1, 1, 1)	8	X		X	X	X	X
5	(2, 0, 0)	6				X		X
6	(2, 1, 0)	24						X
7	(2, 2, 0)	12				X		X
8	(2, 1, 1)	24						X
9	(2, 2, 1)	24						X
10	(2, 2, 2)	8						X
11	(3, 0, 0)	6				X	X	X
12	(3, 1, 0)	24					X	X
13	(3, 2, 0)	24						X
14	(3, 3, 0)	12					X	X
15	(3, 1, 1)	24					X	X
16	(3, 2, 1)	48						X
17	(3, 3, 1)	24					X	X
18	(3, 2, 2)	24						X
19	(3, 3, 2)	24						X
20	(3, 3, 3)	8					X	X

Once the discrete populations $f_{i}^{\text{eq}}$ are known, $g_{i}^{\text{eq}}$ are computed using equation (2.16) as originally proposed by Dubroca *et al.* [32] for DVMs. Adopting the BGK collision model, post-streaming populations are finally computed

2.26 $$h_{i}(x+\xi_{i},t+1)=h_{i}(x,t)-\frac{1}{\tau_{h}}(h_{i}-h_{i}^{\left(\text{eq}\right)})(x,t),$$

where *h* is the population, representing *h* = *f* or *h* = *g*. The above choice of collision model leads to a fixed Prandtl number: Pr = 1. The latter can be adjusted using methodologies based on the kinetic theory of gases (Shakov, Gaussian or ellipsoidal statistical collision models [33,53,54]), or more tightly related—but not restricted—to LBMs such as quasi-equilibrium [39,42,71,72], and two-/multi-relaxation collision models [18,73,74]. This was not implemented for this first study, but in the low-viscosity regime or for negligible heat transfers, it is expected that the error induced by such an approximation is negligible.

In case better stability is required, the relaxation time is made Knudsen-number dependent. This is done by locally computing

2.27 $$\epsilon =\frac{1}{V}\sum_{i=0}^{V-1}\frac{|f_{i}-f_{i}^{\text{eq}}|}{f_{i}^{\text{eq}}},$$

where *ϵ* ≈ Kn according to the Chapman–Enskog expansion at the Navier–Stokes Fourier level [75]. For large departures from the equilibrium state, such that *ϵ* > 10^−2^, the continuum limit assumption does not hold anymore, and the recovery of the macroscopic behaviour of interest is then at risk. To prevent such an issue, one could refine the grid mesh depending on the value of *ϵ*, as proposed by Thorimbert *et al.* [76]. Here, we prefer to damp high-order contributions, as soon as they appear in under-resolved areas of the simulation domain, by adjusting the relaxation time accordingly to the local departure from the equilibrium state: *τ*(*ϵ*) = *τα*(*ϵ*). As a simple *proof of concept*, *α* is chosen as a slowly increasing piecewise constant function of the local Knudsen number *ϵ* which, in the worst case, replaces the populations by their equilibrium value (*ϵ* ≥ 1):

2.28 $$\alpha =\left\{ \begin{array}{lll} 1, & & \epsilon <10^{-2}\\ 1.05, & \;10^{-2}\leq & \epsilon <10^{-1}\\ 1.35, & \;10^{-1}\leq & \epsilon <1\\ 1/\tau , & \; & \epsilon \geq 1 \end{array}\right.$$

where 1/*τ* ≈ 2 in the context of high Reynolds-number flows, which ensures the well-posedness of *α*, i.e. *α* > 1.35 when *ϵ* ≥ 1. This methodology amounts to artificially increasing the viscosity in areas of strong departure from the equilibrium state. This information could also be used to better control the relaxation of high-order moments, similarly to the KBC collision model [77], or to locally activate subgrid scale models [78] and shock capturing techniques [79]. Such investigations will be presented in a future work.

It is finally worth noting that our stabilization technique shares the same philosophy as the so-called entropic filtering approach [80–83]. For the latter, the LB scheme is also stabilized by introducing an artificial dissipation term that depends on the local departure from equilibrium, which can be estimated through several entropic metrics. As an example, the following quadratic entropy estimate

$$\Delta S=\sum_{i=0}^{V-1}\frac{{\left(f_{i}-f_{i}^{\text{eq}}\right)}^{2}}{f_{i}^{\text{eq}}}$$

was used in the comparative study by Gorban & Packwood [83] to dynamically compute either second- or higher-order relaxation times by making them proportional to Δ*S*. The use of the *l*_1_-norm

2.29 $$||\;f-f^{\text{eq}}|{|}_{1}=\sum_{i=0}^{V-1}|f_{i}-f_{i}^{\text{eq}}|,$$

was also evoked by the latter authors, without being investigated in their work. Yet, the normalization by $f_{i}^{\text{eq}}$ is missing from the *l*_1_-norm (2.29), and this prevents the proper evaluation of the departure from equilibrium in terms of Knudsen number. All of this confirms that our stabilization technique cannot be recast in terms of entropic filtering, but instead, it is an innovative way to improve the numerical stability of LBMs through kinetic considerations.

## 3. Macroscopic errors and numerical stability

### (a) Motivation and methodology

In this section, a comprehensive comparison of single population LBMs based on the numerical equilibrium (2.13) is proposed for various velocity discretizations, as well as number of constraints.

It includes lattices composed of 15, 19, 27, 39, 125 and 343 discrete velocities which are spread over three layers of neighbours (table 1). The first three (standard lattices) are of particular interest since they only include one layer of neighbours and are therefore particularly suitable for HPC architectures. Lattices composed of more than a hundred velocities are used as reference, whereas the Q39 is considered as a good trade-off between accuracy and efficiency, hence its use in the current state-of-the-art hybrid LB solver dedicated to the simulation of industry-oriented transonic and supersonic flows [22,44,45].

In addition, an increasing number of constraints are accounted for in the computation of the exponential equilibria (5, 10, 13, 20). They are compiled in appendix B, alongside with their corresponding exponential equilibria and Jacobian matrices. It is worth noting that results corresponding to 14- and 21-moment approaches are not presented since they were found to lead to results very similar to the 13- and 20-moment approaches, respectively. Regarding the model based on 26 constraints, even though it exactly recovers the Navier–Stokes–Fourier equations, it induces a great overhead, as well as convergence issues for the root-finding algorithm. Consequently, it is not studied hereafter.

Since the Lagrange multipliers used for the computation of the numerical equilibrium do not have an analytical expression, a particular methodology—different from standard approaches such as the Chapman–Enskog expansion—needs to be used in order to quantify macroscopic discrepancies with respect to the targeted physics. This can be done by following the methodology proposed by Kornreich *et al.* [50], which was used for the evaluation of macroscopic errors related to the 5-moment approach in the context of LGCA [50], and further considered in the LB framework by Frapolli *et al.* [43]. Concretely, it relies on the computation of the error between the moment of the Maxwellian $M_{pqr}^{\text{MB}}$ and its discrete counterpart $M_{pqr}^{\text{eq}}$

3.1 $$\varepsilon =\frac{|M_{pqr}^{\text{MB}}-M_{pqr}^{\text{eq}}|}{M_{pqr}^{\text{MB}}},$$

with respect to both temperature *T* and the velocity ***u***, or equivalently, the Mach number Ma.

Before moving to the parametric study, it is worth noting that a *necessary but non-sufficient* stability condition is the ability of the root-finding algorithm to obtain an adequate solution to the optimization problem under a certain, as low as possible, number of iterations. If the latter condition is not verified, then the equilibrium cannot be computed and the solver crashes. To maximize the stability of the present approach, several solvers available in the GSL library (https://www.gnu.org/software/gsl/doc/html/multiroots.html) were tested. Among them, those based on Jacobian-free algorithms lead to severe stability issues even for low-Mach numbers. This is the reason why the multivariate Newton–Raphson solver—which requires the computation of the Jacobian of constraints (and its inverse)—was used for all studies proposed in this work.

### (b) Optimal temperature

Contrarily to conventional approaches [14,58,69,70,84], where the reference temperature *T*_0_ is fixed for each velocity discretization, hereafter a more pragmatic path is followed. Indeed, since the present LBM is quadrature-free, *T*_0_ should only be chosen according to the following two points: (1) stable simulations, and (2) minimal macroscopic deviations from the behaviour of interest.

The above considerations are user-dependent and can be adjusted to improve the stability and efficiency of the resulting LBM. For example, by decreasing *T*_0_, it is possible to reach higher Mach numbers for a given freestream velocity, and consequently, to widen the stability range of the LBM. With this idea in mind, we will then only look for optimal temperatures such that *T*_0_ ≤ 1. In addition, we will allow ourselves a *maximal* error margin of ε ≈ 10%, in order to choose the smallest lattice between two velocity discretizations that would have an (almost) equivalent accuracy. The latter choice will eventually lead to huge savings in terms of memory consumption and wall-clock time.

**Figure 1.**
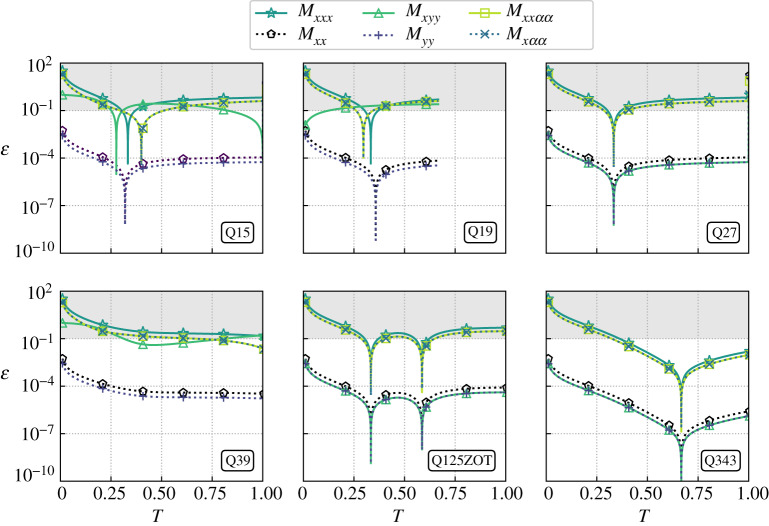
Temperature impact on macroscopic errors ε for the 5-moment approach considering several lattices of increasing size (Ma = 0.01). The grey zone starts at ε = 10%. (Online version in colour.)

To choose the reference temperature, let us focus on macroscopic errors with respect to equilibrium moments that correspond to both convective ($M_{xx}^{\text{eq}}$, $M_{yy}^{\text{eq}}$ and $M_{x\alpha \alpha }^{\text{eq}}$) and diffusive ($M_{xxx}^{\text{eq}}$, $M_{xyy}^{\text{eq}}$ and $M_{xx\alpha \alpha }^{\text{eq}}$) phenomena. Corresponding results are compiled in figure 1 for the 5-moment approach with the Q15, Q19, Q27, Q39, Q125ZOT and Q343 lattices.

Qualitatively speaking, all lattices are stable for the considered range of temperature, but the Q19 and Q27 which become unstable for *T* ≈ 0.7 and 1.0, respectively. The temperature that minimizes macroscopic errors is not necessarily unique, and it depends on the lattice and the moment considered. In addition, errors related to second-order moments are among the lowest ones whatever the lattice considered.

Going into more detail, temperatures that minimize all macroscopic errors at the same time are available for velocity discretizations that are built through tensor products of 1D lattices (Q27, Q125ZOT and Q343). Nevertheless, the latter reference temperatures only correspond to the theoretical ones for the Q27 lattice (*T*_0_ = 1/3). Several temperatures minimize second-, third- and fourth-order macroscopic errors for the Q15 and the Q19, whereas no such temperature is found for the Q39. Nevertheless, its theoretical reference temperature *T*_0_ = 2/3 does lead to acceptable error levels, i.e. close to ε ≈ 10% for 0.5 ≤ *T* ≤ 1.

**Figure 2.**
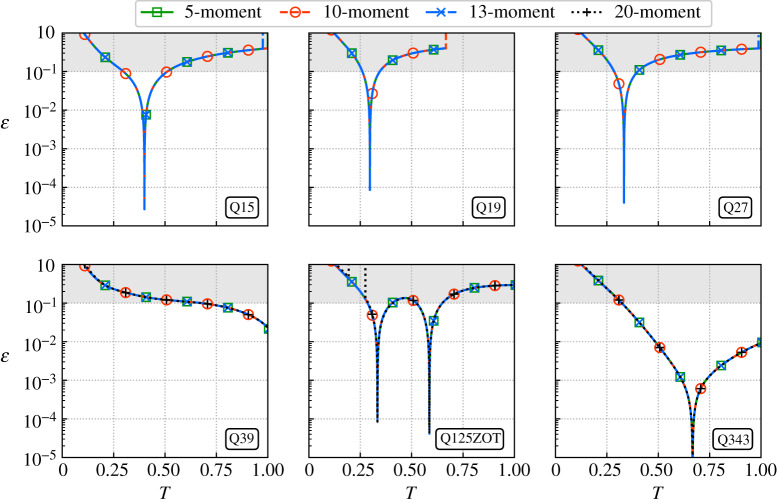
Temperature impact on the error ε of $M_{xx\alpha \alpha }^{\text{eq}}$ for the 5-, 10-, 13- and 20-moment approaches considering several lattices of increasing size (Ma = 0.01). Standard lattices are not stable for the 20-moment approach. (Online version in colour.)

Eventually, it was noticed that the above results (error levels and reference temperatures) remain globally unchanged when the number of constraints was increased from 5 to 20 (figure 2). Hence, only optimal temperatures obtained with the 5-moment approach will be considered for the rest of the study. They are gathered in table 2 for all considered lattices.

**Table 2.** Reference temperatures *T*_0_ that minimize macroscopic errors. For lattices where more than one value was found for the Q15, Q19, Q39 and Q125ZOT, a trade-off had to be found. It consists in either an average of all optimal temperatures (Q15, Q19 and Q39), or the lowest temperature to increase the stability domain (Q125ZOT).

lattice	Q15	Q19	Q27	Q39	Q125ZOT	Q343
*T*_0_	0.35	0.32	1/3	0.6	0.33369	0.66710

### (c) Mach number impact on accuracy and stability

**Figure 3.**
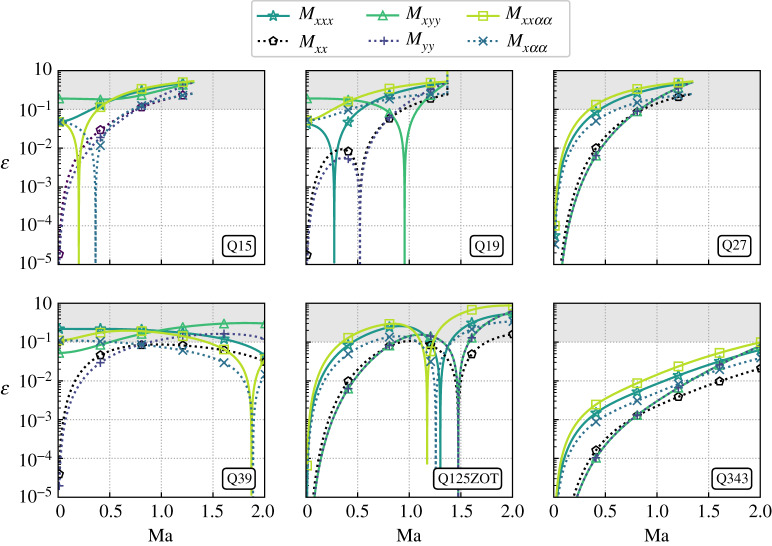
Mach number impact on macroscopic errors ε for the 5-moment approach and *T* = *T*_0_ (see table 2 for their values). The flow propagates along the *x*-axis, and the grey zone starts at ε = 10%. (Online version in colour.)

Let us first focus on the 5-moment approach. Corresponding results are compiled in figure 3 for *T* = *T*_0_ and a flow propagating along the *x*-axis.

First of all, error levels strongly depend on the moment and lattice considered. Globally, they tend towards a plateau when the Mach number increases, but local minima are also encountered for Ma ≠ 0. In addition, lattices based on tensor product of 1D models are the only ones which have minimal errors for all moments when Ma = 0, whereas such a behaviour is only true for second-order moments with the Q15, Q19 and Q39. The largest lattice (Q343) leads to the best accuracy. Regarding stability, even though standard lattices encounter convergence issues with the Newton–Raphson algorithm for Ma ≈ 1.3, they confirm the drastic improvement induced by the use of the numerical equilibrium as compared to its polynomial counterpart. Other lattices do not encounter any convergence issue with the Newton–Raphson algorithm, at least up to Ma = 2.

Up to now, the Q39 seems to be a good compromise in terms of stability and efficiency, but regarding accuracy, error levels can reach 20% for both convective and diffusive terms. While it is possible to balance diffusive errors by restricting the model to the simulation of high Reynolds-number flows, convective errors will still be present and drastically impact the physics. A way to improve the accuracy of such a lattice would be to rely on numerical equilibria based on more constraints. Indeed, it is expected from the statistics point of view that by matching more moments of the Maxwellian, errors related to unconstrained moments will decrease. Nevertheless, one must bear in mind that increasing the number of constraints also makes the optimization problem even more difficult to solve. Hence, it might lead to convergence issues.

**Figure 4.**
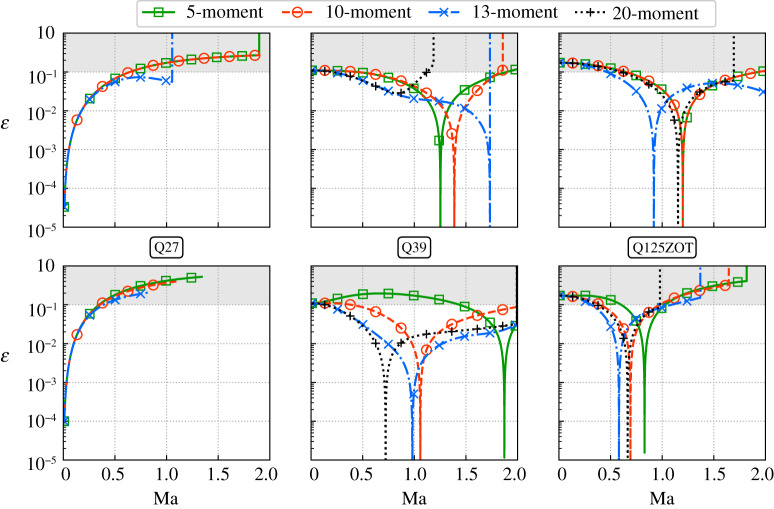
Mach number impact on the error ε of $M_{xx\alpha \alpha }^{\text{eq}}$ for the 5-, 10-, 13- and 20-moment approaches (*T* = *T*_0_). Two different flow orientations are investigated. From top to bottom: diagonal (Ma/3,Ma/3,Ma/3) and *x*-aligned (Ma,0,0). The Q27 encountered severe convergence issues for the 20-moment approach, hence, corresponding results are not plotted. (Online version in colour.)

Henceforth, the impact of the number of constraints (*M* ∈ {5, 10, 13, 20}) is investigated for the Q27, Q39 and 125ZOT lattices. Two flow orientations are further studied in order to better evaluate the stability domain of each LBM. They correspond to a flow propagating along: the *x*-axis (Ma, 0, 0), and the diagonal (Ma/3, Ma/3, Ma/3). In addition, we will only focus on the error related to $M_{xx\alpha \alpha }^{\text{eq}}$ because for the most constrained case (*M* = 20), third- and lower-order moments are error-free. Corresponding results are plotted in figure 4. It is interesting to note that the two configurations lead to different error levels as well as stability domains whatever the lattice considered. In addition, increasing the number of constraints does seem to decrease error levels related to $M_{xx\alpha \alpha }^{\text{eq}}$, at least, when the Newton–Raphson does not encounter convergence issues. The Q27 lattice is the velocity discretization that suffers the most from the increase of *M*, whereas high-order lattices remain stable whatever the configuration considered for Ma ≤ 1. Interestingly, the Q39 competes with the Q125ZOT when *M* ≥ 10 for both flow orientations. Eventually, it is for the 13-moment approach that the Q39 clearly outperforms the Q125ZOT by reaching an excellent trade-off in terms of stability and accuracy (less than 5% error levels for most of the Mach number range).

For all of these reasons, the rest of the paper will be dedicated to the study of the numerical properties and efficiency of the 13-moment based D3Q39-DDF-LBM (§4 and 5, respectively).

## 4. Numerical tests

### (a) One-dimensional Riemann problem

As a first validation, the generation and propagation of both rarefaction and shock waves is investigated through the simulation of a 1D Riemann problem, also known as Sod shock tube [85]. The following density and pressure jumps are considered (*ρ*_*L*_/*ρ*_*R*_, *P*_*L*_/*P*_*R*_) = (8, 10), and set up through: (*ρ*_*L*_, *T*_*L*_) = (8, 0.75) and (*ρ*_*R*_, *T*_*R*_) = (1, 0.6). Subscripts *L* and *R* stand for the left and the right states, respectively. The gas is considered at rest for both states, and the simulation domain is discretized along the *x*-direction using only *L*_*x*_ = 400 grid points.

**Figure 5.**
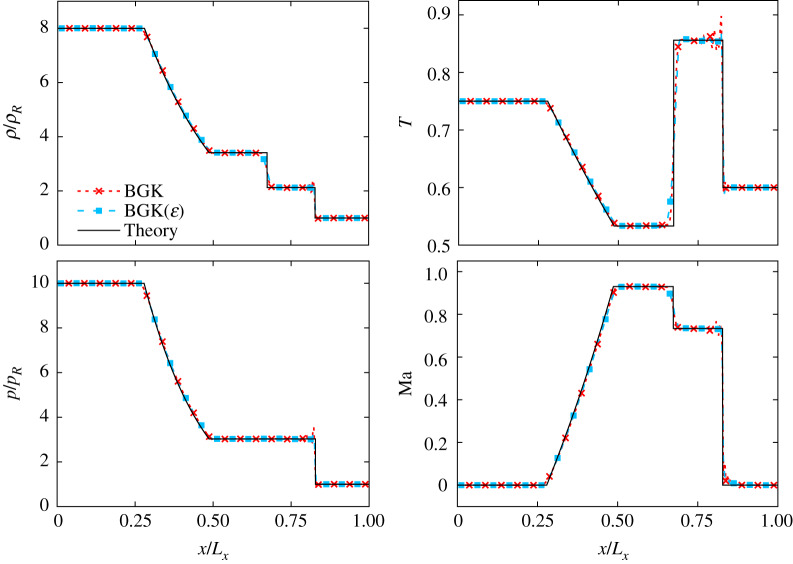
Sod shock tube for a diatomic gas (specific heat ratio *γ*_*r*_ = 7/5). Results obtained with *L*_*x*_ = 400 points using the standard BGK operator, and our version [BGK(*ϵ*)], are compared against theoretical curves, for *τ*_*f*_ = 0.57 and 0.5, respectively. From top left to bottom right: normalized density, temperature, pressure profiles and local Mach number evolution. (Online version in colour.)

Corresponding results are compiled in figure 5 for both the standard BGK operator and our dynamic version based on equation (2.28). They prove the ability of the proposed model to simulate the generation and propagation of rarefaction and shock waves in a diatomic gas. Simulations are stable for relatively low values of the relaxation time (*τ*_*f*_ = 0.57, Pr = 1), even with the standard BGK operator, while LBMs based on a polynomial discrete equilibrium usually require a large number of discrete velocities (e.g. 81 in 2D [73]), more robust collision models [16,17] or numerical discretizations [86], in order to achieve similar results. Interestingly, the Knudsen number based relaxation times drastically improve the stability of the present model, allowing the simulation of the 1D Riemann problem in the zero-viscosity limit (*τ*_*f*_ = 0.5, Pr = 1), with an accuracy that competes with LBMs coupled with shock capturing techniques [87].

**Figure 6.**
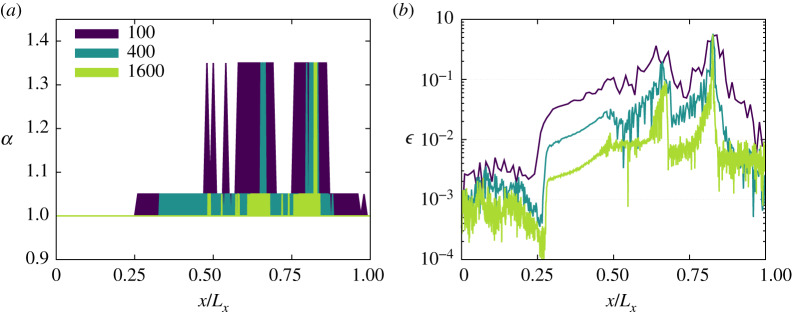
Sod shock tube for a diatomic gas (specific heat ratio *γ*_*r*_ = 7/5). Behaviour of the kinetic parameter *α* (*a*) and sensor *ϵ* (*b*) for three mesh sizes: *L*_*x*_ = 100, 400 and 1600 points along the *x*-axis. (Online version in colour.)

The behaviour of this kinetic sensor is investigated in more detail in figure 6. Interestingly, it locally introduces additional viscosity in regions of the simulation domain where (1) under-resolved conditions, and (2) abrupt changes of macroscopic quantities are encountered. Furthermore, by refining the grid mesh, the kinetic parameters self-adjust and focus the addition of viscosity on both discontinuities (contact and shock). This behaviour is similar to the one observed with shock-capturing techniques, as discussed in the context of high-order LBMs by Coreixas [87]. Eventually, while a (non-local) space averaging was required in the latter work to smooth the normalized pressure Laplacian used as a shock sensor, the present (local) parameter *α* allows the smoothing of the input provided by *ϵ* without impacting the parallel performances of our LBM.

### (b) Supersonic flow past a 2D aerofoil

The next validation test consists of the simulation of the flow past a NACA0012 aerofoil in the supersonic regime, and for a relatively high Reynolds number (Re = 10^4^). Before moving to the results, it is worth noting that the standard BGK-LBM based on the equilibrium (2.13) led to stable simulations for Mach and Reynolds numbers up to 1.2 and 7500, respectively. These parameters are however insufficient for a comparison against data available in the literature [42,88], which are provided for (Ma, Re) = (1.5, 10^4^). Hence, the dynamic relaxation time based on the *α* function (2.28) was used to further extend the stability range of the present approach. The simulation domain is defined as [*n*_*x*_, *n*_*y*_, *n*_*z*_] = [8*C*, 8*C*, 1] with *C* = 350 points, *n*_*j*_ being the number of points in the *j* direction (*j* = *x*, *y* or *z*), and centred around the leading edge of the aerofoil. While the freestream conditions are imposed on the left, top and bottom boundary conditions of the domain (by imposing $f_{i}=f_{i}^{\text{eq}}$ and $g_{i}=g_{i}^{\text{eq}}$), a (first-order) Neumann boundary condition is imposed at the (right) outlet. The no-slip boundary condition is imposed on the aerofoil using the half-way bounce-back methodology [4].

**Figure 7.**
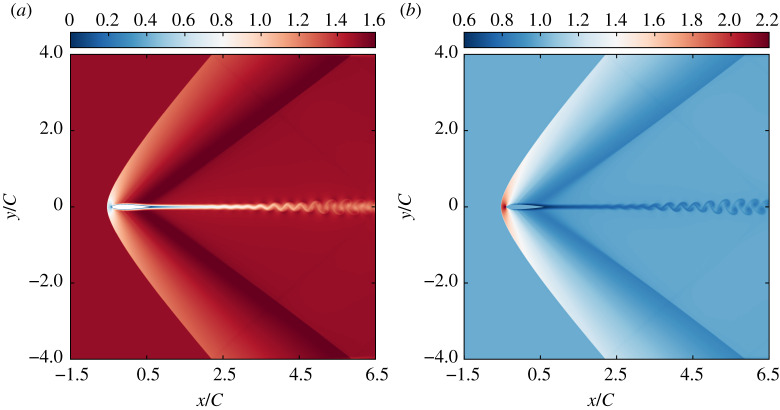
NACA0012 aerofoil at Ma = 1.5 and Re = 10^4^ using *C* = 350 points: local Mach number (*a*) and density (*b*) fields. (Online version in colour.)

**Figure 8.**
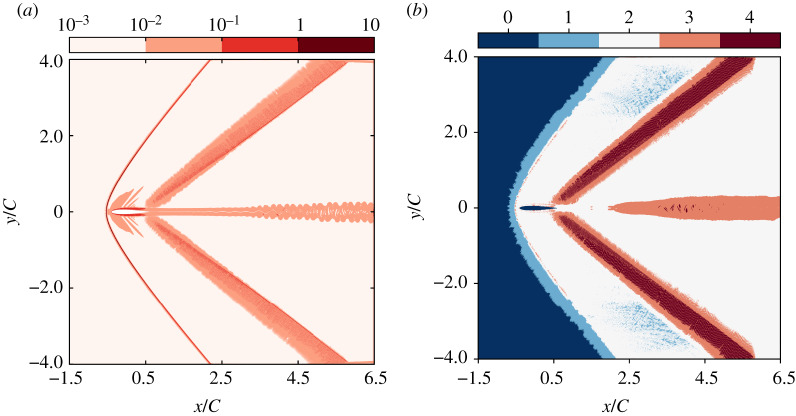
NACA0012 aerofoil at Ma = 1.5 and Re = 10^4^ using *C* = 350 points: local Knudsen number *ϵ* (*a*), and number of iterations of the Newton–Raphson solver (*b*). (Online version in colour.)

As a first insight on the physical phenomena related to this numerical validation, the local Mach number and density fields are plotted in figure 7. They highlight the main features of the flow: (1) primary strong bow shock upward the leading edge, (2) secondary weak shock close to the trailing edge, and (3) vortex shedding downward the aerofoil. This qualitatively confirms the good behaviour recovered thanks to the new equilibrium (2.13), as compared to results published in [43,88]. To investigate the numerical properties of the present approach, the local Knudsen number *ϵ*, as well as the number of Newton-Raphson iterations are plotted in figure 8. Interestingly, the kinetic sensor *ϵ* is only activated close to the aforementioned features, which allows us to properly classify them in terms of departure from the equilibrium. This observation serves as a justification for the fact that the adaptive BGK operator leads to stable simulations through a local increase of the kinematic viscosity adapted to the flow features: high increase for both strong and weak shocks, moderate close to the aerofoil, low in its wake, none in the rest of the domain. This indicates that one of the possible outcomes of such a sensor would be a better control over areas of the simulation domain where shock-capturing techniques, and/or subgrid scale models should be activated. In addition, it is confirmed that the number of Newton–Raphson solver iterations required to impose the correct 13 moments is relatively low. In this example, it is four at most, less than two in average, and zero over a wide area, as the Lagrange multipliers inherited from the previous time step remain satisfying. It is also interesting to point out that the number of iterations follows the main flow features. Surprisingly, the highest number of iterations is not observed in the region where the highest departure from equilibrium is reported (bow shock), but instead, it is needed close to the secondary shock where the highest Mach number is encountered. This information indicates that a potential stability issue could arise from this part of the simulation domain. Hence, it is hypothesized that the number of iterations could be used to further activate last-resort stabilization techniques if need be.

**Figure 9.**
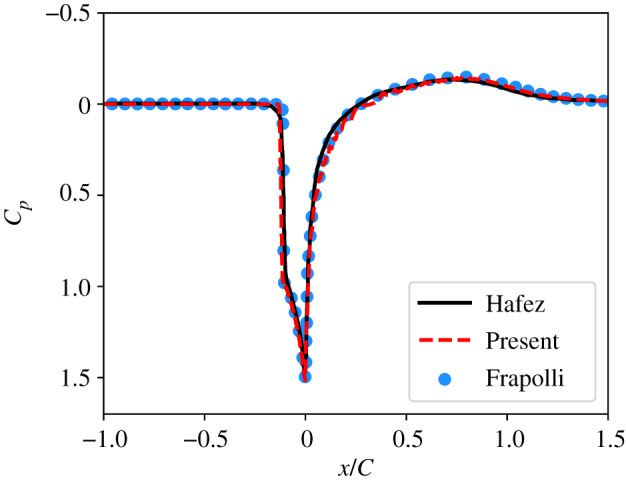
Comparison of *C*_*p*_ distribution around the NACA0012 aerofoil at Ma = 1.5 and Re = 10^4^ using *C* = 350 points. Data are compiled from Hafez *et al.* [88] and Frapolli *et al.* [43]. (Online version in colour.)

Quantitatively speaking, the comparison of pressure coefficient profiles [$C_{p}=(p-p_{\infty })/0.5\rho_{\infty }u_{\infty }^{2}$] in figure 9 clearly shows that our stabilization technique does not impact the level of accuracy reached by the present approach. More precisely, the width of the bow shock is fairly similar to reference solutions despite the equilibration of populations that is imposed in this particular region of the simulation domain. In addition, even if the bounce-back methodology leads to small oscillations close to the walls due to its inherent stair-case approximation, the parietal *C*_*p*_ profile (0 ≤ *x*/*C* ≤ 1) is also in agreement with reference studies. All in all, considering the relatively coarse grid mesh—as compared to [43] where *C* = 800 grid points were used for the 343-shifted-velocity 5-constraints method—and the very simple no-slip boundary condition used in this study, these results are very encouraging. Indeed, accounting for (1) the speedup induced by the use of the D3Q39 lattice instead of the D3Q343, and (2) the fact that stable and accurate results are obtained with *C* = 350 (instead of *C* = 800), the present approach decreases the overall CPU time and memory consumption required to run this simulation by two orders of magnitude as compared to the more standard approach [43]. Interestingly, it is done without relying on shifted versions of the D3Q39 that would further shift its stability domain around the predefined shifted velocity, as demonstrated by Hosseini *et al.* [89] and further investigated in appendix C. It is eventually worth noting that stable simulations were obtained up to Re = 10^9^ in under-resolved conditions (*C* = 200 points). This confirms the excellent properties of our Knudsen number based stabilization technique.

### (c) Supersonic flow around a sphere

Hereafter, the 3D behaviour of our model is investigated in the low-Reynolds, supersonic regime. This is done simulating a supersonic flow past a sphere for a low-Reynolds number Re = 300, and a range of Mach numbers 1.3 ≤ Ma ≤ 1.6. This kind of flow is of particular interest for the aerospace industry, and more precisely, to understand the dynamics of alumina particles that are released from solid rocket motors, and which are believed to be related to the generation of huge acoustic waves during the launching phase [90].

A cubic domain [*n*_*x*_, *n*_*y*_, *n*_*z*_] = [20*R*, 20*R*, 20*R*] is used for the simulation of the flow past a sphere of radius *R*. The latter domain is centred around the sphere. Freestream conditions are applied on all sides but the outlet where a (first-order) Neumann boundary condition is imposed. Once again, the no-slip boundary condition is imposed on the sphere using the half-way bounce-back methodology. Due to the low value of the Reynolds number, it was chosen to use *R* = 15 points meaning that the sphere diameter is discretized using *D* = 30 points. The latter value was found to be sufficient to study the impact of the Mach number on the main characteristics of the flow. Due to the high values of the Mach number, all simulations are performed using the kinetic based stabilizer.

**Figure 10.**
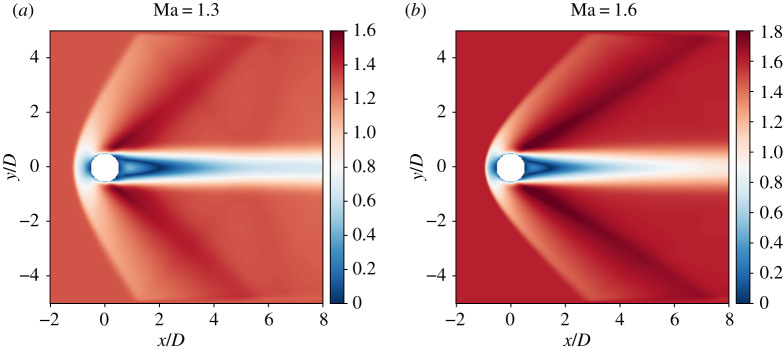
Instantaneous visualization of the 3D supersonic flow past a sphere: Re=300 and two different Mach numbers. The main features of the flow are recovered: bow shock, steady flow, and decrease of the shock stand-off distance *L*_*s*_ as Ma increases. (Online version in colour.)

**Figure 11.**
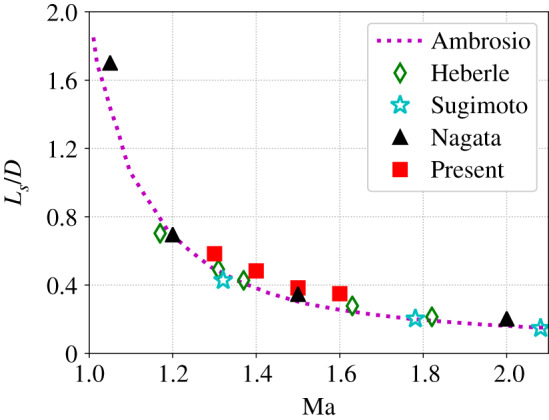
Comparison of shock stand-off distance *L*_*s*_ against experimental data (Sugimoto *et al.* compiled by Hida [91] and Heberle *et al.* [92]), models [93] and numerical simulations [90]. (Online version in colour.)

Figure 10 compiles instantaneous snapshots of the Mach number around the sphere for Re = 300 and 1.3 ≤ Ma ≤ 1.6—where the latter value was identified as the upper limit in terms of numerical stability (local Mach number of Ma ≈ 1.9), which roughly corresponds to the convergence limit of the Newton–Raphson solver (figure 4). Qualitatively speaking, the influence of Ma seems to be correctly reproduced by our model. More precisely, for the present values of Ma and Re, all flows are steady, a bow shock forms at a distance *L*_*s*_ from the sphere, and its curvature increases with the Mach number. In addition, the shock stand-off distance *L*_*s*_ decreases when Ma increases, as observed by Nagata *et al.* [90]. The latter quantity is studied in more detail in figure 11 where it is compared against a large set of data based on experiments (Sugimoto *et al.* compiled by Hida [91] and Heberle *et al.* [92]), models [93] and numerical simulations [90]. Despite a slight overestimation, the right tendency is obtained with the present method. This is very promising considering the fact that a relatively coarse mesh and the very simple halfway bounce back rule were used.

## 5. Implementation and efficiency

Common LB schemes are characterized by a separation between a local collision step, which characterizes the physical properties of the model, and a generic, non-local streaming step. The collision step is usually carried out by manipulating the component of the populations that deviates from equilibrium, through a linear term, as in the straightforward BGK model [49], or a more general expression, as in multiple-relaxation-time approaches [4]. A crucial importance is therefore attributed to the explicit calculation of the equilibrium term, as a function of the macroscopic variables density, velocity and temperature. As a matter of fact, the expression of the equilibrium distribution can be considered to entirely determine the physics expressed by the model [94,95], if and only if the different non-equilibrium contributions are relaxed to recover the correct transport coefficients of the macroscopic equations of interest (see [96] for a systematic approach in the context of fluid mechanics).

While LB models frequently approximate the equilibrium through an explicitly constructed polynomial expression [4,5], the present paper follows the path of DVMs and adopts a computationally more expensive approach of exponential-of-polynomial expression with iteratively computed coefficients, as extensively discussed in [29,30]. Here, the equilibrium is computed through the following process described in §2d where *V* = 39 and *M* = 13.

The computational expense of this algorithm is dominated by the inversion of the Jacobian *J*. It can be mentioned that while the construction of *J* itself is costly as well, the number of terms to be computed can be reduced from a total of 13 × 13 = 169 terms to only 48, due to sparsity and redundancy of *J*. It is interesting to point out that in spite of the added complexity, the proposed model remains entirely local. It therefore increases the number of arithmetic operations to be executed per cycle, but adds little to the total number of memory accesses, which favourably matches the expectations of modern computing. In particular, such properties can be considered in favour of the use of hardware accelerators.

For the purposes of this article, the model was implemented as a self-standing CPU code, written in the C++ language, and a self-standing GPU code written in CUDA. These codes cannot be compared in a straightforward manner to 5-moment entropic models, as these require the use of a D3Q343 lattice to achieve supersonic results as those presented in §4. Comparisons with models based on a polynomial equilibrium are just as difficult, as the current literature provides no evidence that such an approach could allow us to simulate non-trivial supersonic flows, except by introducing a substantially more complex collision model [16,87]. A comparison is nevertheless presented with a BGK model based on an equilibrium derived through the Hermite polynomial expansion up to the fourth order, which is the minimum truncation order required to fully recover the macroscopic behaviour of the Navier–Stokes–Fourier equations [14]. In addition, comparisons are made based on double precision computations. The reason for this is twofold: (1) GPUs are dedicated to single precision computations and adopting the latter precision would bias the comparison, and (2) single precision computations can lead to accuracy issues when simulating acoustically related phenomena because their amplitude is, roughly speaking, two to three orders of magnitude smaller than those related to hydrodynamics. In the end, while the numbers cannot be taken as a competitive comparison (the polynomial model cannot solve the tests of §4 with the D3Q39 lattice [14,70]), they provide a general understanding of the overall performance of our model.

The polynomial model is executed on the D3Q39 lattice and uses a double set of population to mimic the double-population approach of our model to vary the adiabatic expansion coefficient [32,43,52,97]. The performance is measured in terms of MLUPS (million lattice updates per second). For the two 13-moment codes, the benchmark is the NACA0012 aerofoil of §4 with a 2048 × 2048 resolution (i.e. *C* = 256). The polynomial code was implemented in the Palabos library [98], and executed for a generic, subsonic flow problem with the same number of nodes. The CPU hardware is an Intel Core i7-8700 CPU at 3.2 GHz with 6 cores, where all 6 cores are exploited through OpenMP (13-moment code) or MPI (polynomial code). The GPU code runs on a single Nvidia GeForce GTX 1080 Ti with 11Gb of central memory. The results are presented in the following table:

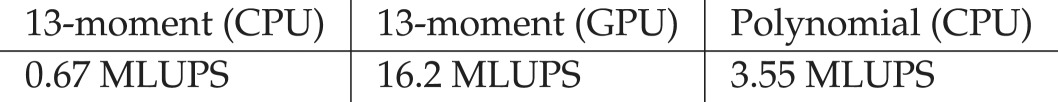

The table shows that the 13-moment CPU code exhibits only a fivefold speed-down with respect to the fourth-order polynomial code, a price that is cheap to pay for the gain of proper supersonic physics. In addition, the 13-moment approach leads to CPU performances that are typical of standard NSF solvers [48], which further confirms the viability of the proposed approach for everyday use. Interestingly, a more than 20-fold speedup, compared to a multi-core CPU implementation, is obtained with the help of a relatively straightforward GPU code, providing more than satisfying performance for the simulation of realistic configurations in an industrial context. To give an example, the NACA0012 benchmark of §4 (i.e. *C* = 350) was fully carried out on a single GPU and reached the full duration of 15 000 iterations in less than three hours.

## 6. Discussion and outlook

A new type of quadrature free LBM was introduced in the context of compressible flow simulation. It relies on a numerical equilibrium (extensively used in the context of DVMs), that can reproduce an arbitrary number of moments of the Maxwell–Boltzmann distribution over a large range of velocity discretizations. The latter aspect is of particular interest in order to recover the targeted macroscopic behaviour using velocity discretizations as small as possible. In this paper, several lattices composed of 15, 19, 27, 39, 125 and 343 velocities were implemented and compared against each other for different sets of macroscopic constraints (mostly 5, 10, 13 and 20).

After an in-depth investigation of the accuracy and stability of all the above LBMs, the D3Q39 lattice along with the 13-moment based equilibrium were chosen to accurately simulate flows in the supersonic regime. The latter choice is a tremendous improvement as compared to previous similar methods that relied on 343 velocities. Also, the computational cost of the proposed solution procedure is shown to remain in acceptable limits, with a speed down of a factor 5 only compared to a method with polynomial equilibrium (which cannot reproduce the same compressible physics). Nevertheless, the computational overhead can be compensated as the method is shown to allow substantial speedups through the use of accelerators, as shown through a 20-fold speedup using an Nvidia GPU. This guarantees the viability of the 13-moment approach in an industrial context.

In addition, we proposed a kinetic sensor which is used to locally increase the viscosity according to the departure from equilibrium. Considering an extremely simple formulation of this sensor, stable and accurate simulations were obtained for a 1D Riemann problem, a high-Reynolds number flow past a NACA0012 aerofoil, and a low-Reynolds one over a sphere, with the latter two in the supersonic regime. This proves that, in the context of LBMs, one does not need to rely on the entropic formulation in order to achieve both stable and accurate simulations via the exponential equilibrium. Consequently, this work confirms the importance of the numerical equilibrium introduced by DVMs, as compared to the collision model, since the latter can always be supplemented by any kind of subgrid scale model coupled with a shock capturing technique in order to achieve stable simulations in the supersonic regime. Interestingly, the preliminary study presented in Appendix C shows that by including the concept of shifted lattices in the present methodology, the latter is no longer bounded to Ma ≤ 2 for the D3Q39, and higher Mach numbers can easily be reached.

In the view of the general nature of LBMs based on numerical equilibria, it seems quite natural to think that the present approach could be extended to simulate other types of flows (multiphase, magnetohydrodynamic, semi-classical, relativistic, etc.) and physics (electromagnetism, quantum systems, etc.), by simply changing the constraints used to compute the numerical equilibrium. This assumption is corroborated by DVMs that were proposed, in the late 1990s and early 2000s, for the simulation of radiative phenomena [28,36]. In the context of LBMs, this would be particularly suitable for semi-classical [99] and relativistic [100] LBMs, where a large number of constraints must be satisfied to recover the macroscopic behaviour of interest, and this imposes the use of very large lattices.

Regarding the kinetic sensor, even though it could be replaced by any other stabilization technique, it possesses several interesting properties. First, it only depends on the departure from equilibrium, and can correspondingly be extended to any type of physics. Second, it can also be used to identify under-resolved areas where either a stabilization technique (subgrid scale models and/or shock capturing approach) or mesh refinement are required. For the former, the sensor would definitely offer a better control on the different scales impacted by these stabilization techniques—which is usually their main flaw [79].

Eventually, in order to make our work widely accessible, we intend to make the present models available in the near future for both CPU and GPU architectures, in the context of a release of the open-source Palabos code [98].

## Figures and Tables

**Figure 12.**
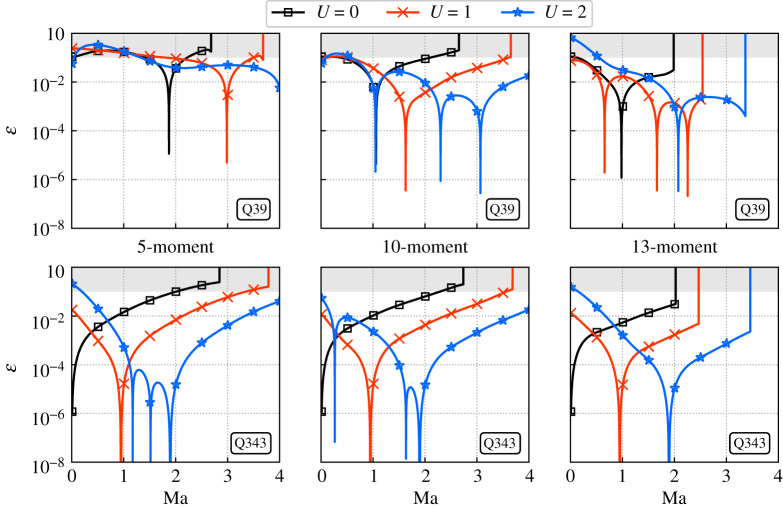
Impact of the shifted velocity *U* on the error ε of $M_{xx\alpha \alpha }^{\text{eq}}$ for the 5-, 10- and 13-moment approaches (*T* = *T*_0_). Two lattices are considered (from top to bottom): the Q39 and Q343 lattices. The flow propagates along the *x*-axis. (Online version in colour.)

## Data accessibility

This manuscript has no further supporting data.

## Appendix A. Derivation of the discrete equilibrium through MEP

By definition, the *discrete* Maxwellian is the quantity that minimizes the *H*-functional [55]

A 1 $$H=\sum_{i}\;f_{i}\left[\ln\left(\frac{f_{i}}{a}\right)\right],$$

under the constraints of mass, momentum and energy conservations

A 2 $$G_{\rho }=\sum_{i}\;f_{i}-\rho =0,\;G_{\rho u_{\alpha }}=\sum_{i}\;f_{i}c_{i,\alpha }-\rho u_{\alpha }=0,\;G_{\rho E}=\sum_{i}\;f_{i}c_{i}^{2}-2\rho E=0.$$

To minimize *H*, one simply needs to solve the system of equations

A 3 $$\forall i,\;\nabla_{f_{i}}H=\frac{\partial H}{\partial f_{i}}=0.$$

Nevertheless, the solution of equation (A 3) does not necessarily satisfy the macroscopic constraints $G_{\rho }=G_{\rho u_{\alpha }}=G_{\rho E}=0$. In order to further take into account these constraints in the minimization of equation (A 1), one may rely on the Lagrange multipliers (see [101] for their intuitive interpretation), which are defined in the present case as

A 4 $$\forall i,\;-\frac{\partial H}{\partial f_{i}}=\lambda_{\rho }\frac{\partial G_{\rho }}{\partial f_{i}}+\lambda_{\rho u_{\alpha }}\frac{\partial G_{\rho u_{\alpha }}}{\partial f_{i}}+\lambda_{\rho E}\frac{\partial G_{\rho E}}{\partial f_{i}}.$$

A simple way to satisfy this condition is to consider the Lagrange function

A 5 $$L^{H}=H+\lambda_{\rho }G_{\rho }+\lambda_{\rho u_{\alpha }}G_{\rho u_{\alpha }}+\lambda_{\rho E}G_{\rho E}$$

which is related to the minimization of *H* (A 1) under the constraints (A 2). This Lagrange function *L*^*H*^ depends on all populations *f*_*i*_ as well as Lagrange multipliers ($\lambda_{\rho },\lambda_{\rho u_{\alpha }},\lambda_{\rho E}$), whereas *H* is only a function of *f*_*i*_. Hence, it can be shown that the minimization of *H* under constraints $G_{\rho }=G_{\rho u_{\alpha }}=G_{\rho E}=0$ is equivalent to the minimization of the Lagrange function, i.e.

A 6 $$\left\{ \begin{array}{l} \nabla_{f_{i}}(H+\lambda_{\rho }G_{\rho }+\lambda_{\rho u_{\alpha }}G_{\rho u_{\alpha }}+\lambda_{\rho E}G_{\rho E})=0\\ G_{\rho }=G_{\rho u_{\alpha }}=G_{\rho E}=0 \end{array}\right.⟺\nabla_{f_{i},\lambda }L^{H}=0,$$

since

$$\partial_{\lambda_{\rho }}L^{H}=G_{\rho }=0,\;\partial_{\lambda_{\rho u_{\alpha }}}L^{H}=G_{\rho u_{\alpha }}=0,\;\partial_{\lambda_{\rho E}}L^{H}=G_{\rho E}=0.$$

Interestingly, the condition $\nabla_{f_{i}}L^{H}=0$ leads to the following exponential form of the equilibrium state:

A 7 $$f_{i}^{\text{eq}}=a\exp[-(1+\lambda_{\rho }+\lambda_{\rho u_{\alpha }}c_{i,\alpha }+\lambda_{\rho E}c_{i}^{2})],$$

due to the fact that $\nabla_{f_{i}}H=\ln f_{i}+1$.

Applying the same steps with the general set of constraints (2.12), one obtains the following (generalized) Lagrange function:

A 8 $$L_{pqr}^{H}=H+\sum_{p,q,r}\lambda_{M_{pqr}^{\text{MB}}}G_{pqr}.$$

The condition $\nabla_{f_{i}}L_{pqr}^{H}=0$ eventually leads to

A 9 $$f_{i}^{\text{eq}}=a\exp\left[-\left(1+\sum_{p,q,r}\lambda_{M_{pqr}^{\text{MB}}}\xi_{x}^{p}\xi_{y}^{q}\xi_{z}^{r}\right)\right].$$

It is worth noting that the above form of the numerical equilibrium is not unique and, obviously, depends on the scaling factor *a*. To the best of our knowledge, the latter cannot depend on the velocity discretization but it can be space- and/or time-dependent [55]. Usually, *a* = 1 for DVMs [29], while the work by Frapolli *et al.* [41] relies on *a* = *ρw*_*i*_ (*T*) where *w*_*i*_ (*T*) are temperature-dependent quadrature weights. In the present context, we chose to underline (1) the quadrature-free property of our approach by discarding quadrature-weights, (2) the fact that we are applying this methodology to the simulation of fluid flows by keeping the density in the definition of the numerical equilibrium through *a* = *ρ*. The latter choice eventually leads to the numerical equilibrium (2.13). Whereas the latter choice does not have a direct impact on imposed moments, it may lead to different stability domains for the root-finding algorithm. This aspect is under investigation, and corresponding results will be presented in a forthcoming paper.

## Appendix B. Details of the numerical models

**(a) 5-moment model**

The five unknowns *λ*_*k*_, *k* = 0…4 are labelled with two indices, where the first corresponds to the order of the attached moment,

B 1 $$\lambda_{k}=(\lambda_{0},\lambda_{1,0},\lambda_{1,1},\lambda_{1,2},\lambda_{2}).$$

They fully determine the value of the equilibrium:

B 2 $$f_{i}^{\text{eq}}=\rho \exp\left[-(1+\lambda_{0}+\lambda_{1,\alpha }\xi_{i\alpha }+\lambda_{2}\xi_{i\kappa }\xi_{i\kappa })\right].$$

The parameters *λ*_*k*_ must be determined to match the set of constraints of equation (2.12), expressed in this case by five constraints *G*_*k*_ = 0, *k* = 0…4, which are labelled with the same index system as the *λ*_*k*_ in equation (B 1):

B 3 $$\begin{array}{rl} & G_{0}=\sum_{i}\;f_{i}^{\text{eq}}-\rho \;\text{(1 constraint)} \end{array}$$

B 4 $$\begin{array}{rl} & G_{1,\alpha }=\sum_{i}\;f_{i}^{\text{eq}}\xi_{i\alpha }-\rho u_{\alpha }\;\text{(3 constraints)} \end{array}$$

B 5 $$\begin{array}{rl} \text{and}\;\; & G_{2}=\sum_{i}\;f_{i}^{\text{eq}}\xi_{i\kappa }\xi_{i\kappa }-2\rho E\;\text{(1 constraint)}. \end{array}$$

The 5 × 5 Jacobian *J*_*kl*_ = ∂*G*_*k*_/∂*λ*_*l*_ is given as follows:

**(b) 13-moment model**

The 13 unknowns *λ*_*k*_, *k* = 0…12 are labelled using the same convention as in the 5-moment model, equation (B 1):

B 6 $$\lambda_{k}=(\lambda_{0},\lambda_{1,0},\lambda_{1,1},\lambda_{1,2},\lambda_{2,00},\lambda_{2,01},\lambda_{2,02},\lambda_{2,11},\lambda_{2,12},\lambda_{2,22},\lambda_{3,0},\lambda_{3,1},\lambda_{3,2}).$$

They fully determine the value of the equilibrium:

B 7 $$f_{i}^{\text{eq}}=\rho \exp\left[-(1+\lambda_{0}+\lambda_{1,\alpha }\xi_{i\alpha }+\lambda_{2,\alpha \beta }\xi_{i\alpha }\xi_{i\beta }+\lambda_{3,\alpha }\xi_{i\alpha }\xi_{i\kappa }\xi_{i\kappa })\right],$$

where the repeated sum of the second-order term, *λ*_2,*αβ*_*ξ*_*iα*_*ξ*_*iβ*_ involves only the 6 indices present in (B 6), for reasons of symmetry. The parameters *λ*_*k*_ must be determined to match the set of constraints of equation (2.12), expressed in this case by 13 constraints *G*_*k*_ = 0, *k* = 0…12, which are labelled with the same index system as the *λ*_*k*_ in equation (B 6). The first four constraints *G*_0_, *G*_1,*α*_ are the same as in the 5-moment model, equations (B 3) and (B 4), and the remaining constraints are:

B 8 $$G_{2,\alpha \beta }=\sum_{i}\;f_{i}^{\text{eq}}\xi_{i\alpha }\xi_{i\beta }-\rho (u_{\alpha }u_{\beta }+T\delta_{\alpha \beta })\;\text{(6 constraints)}$$

and

B 9 $$G_{3,\alpha }=\sum_{i}\;f_{i}^{\text{eq}}\xi_{i\alpha }\xi_{i\kappa }\xi_{i\kappa }-2\rho u_{\alpha }(E+T)\;\text{(3 constraints)}.$$

The 13 × 13 Jacobian *J*_*kl*_ = ∂*G*_*k*_/∂*λ*_*l*_ is explicited as follows:

Regarding the 10-moment approach, it flows from the above formulas by simply imposing *λ*_3,*α*_ = 0 in (C 7), disgarding constraints (B 9) as well as the corresponding last three rows/columns of the above Jacobian matrix.

**(c) 20-moment model**

The 20 unknowns *λ*_*k*_, *k* = 0…19 are labelled using the same convention as in the 5-moment model, equation (B 1):

B 10 $$\begin{array}{rl} \lambda_{k} & =(\lambda_{0},\lambda_{1,0},\lambda_{1,1},\lambda_{1,2},\lambda_{2,00},\lambda_{2,01},\lambda_{2,02},\lambda_{2,11},\lambda_{2,12},\lambda_{2,22},\\ & \;\lambda_{3,000},\lambda_{3,001},\lambda_{3,002},\lambda_{3,011},\lambda_{3,012},\lambda_{3,022},\lambda_{3,111},\lambda_{3,112},\lambda_{3,122},\lambda_{3,222}). \end{array}$$

They fully determine the value of the equilibrium:

B 11 $$f_{i}^{\text{eq}}=\rho \exp\left[-(1+\lambda_{0}+\lambda_{1,\alpha }\xi_{i\alpha }+\lambda_{2,\alpha \beta }\xi_{i\alpha }\xi_{i\beta }+\lambda_{3,\alpha \beta \gamma }\xi_{i\alpha }\xi_{i\beta }\xi_{i\gamma })\right],$$

where the repeated sum of the second- and the third-order term involve only indices present in (B 10) (6 terms at second-order, 10 terms at third-order), for reasons of symmetry. The parameters *λ*_*k*_ must be determined to match the set of constraints of equation (2.12), expressed in this case by 20 constraints *G*_*k*_ = 0, *k* = 0…19, which are labelled with the same index system as the *λ*_*k*_ in equation (B 10). The first four constraints *G*_0_, *G*_1,*α*_ are the same as in the 5-moment model, equations (B 3) and (B 4), the following six constraints *G*_2,*αβ*_ are the same as in the 13-moment model, equation (B 8), and the remaining constraints are:

B 12 $$G_{3,\alpha \beta \gamma }=\sum_{i}\;f_{i}^{\text{eq}}\xi_{i\alpha }\xi_{i\beta }\xi_{i\gamma }-\rho (u_{\alpha }u_{\beta }u_{\gamma }+Tu_{\alpha }\delta_{\beta \gamma }+Tu_{\beta }\delta_{\gamma \alpha }+Tu_{\gamma }\delta_{\alpha \beta })\;\text{(10 constraints)}.$$

The 20 × 20 Jacobian *J*_*kl*_ = ∂*G*_*k*_/∂*λ*_*l*_ is given as follows:

**(d) 26-moment model**

The 26 unknowns *λ*_*k*_, *k* = 0…25 are labelled using the same convention as in the 5-moment model, equation (B 1):

B 13 $$\begin{array}{rl} \lambda_{k} & =(\lambda_{0},\lambda_{1,0},\lambda_{1,1},\lambda_{1,2},\lambda_{2,00},\lambda_{2,01},\lambda_{2,02},\lambda_{2,11},\lambda_{2,12},\lambda_{2,22},\\ & \;\lambda_{3,000},\lambda_{3,001},\lambda_{3,002},\lambda_{3,011},\lambda_{3,012},\lambda_{3,022},\lambda_{3,111},\lambda_{3,112},\lambda_{3,122},\lambda_{3,222},\\ & \;\lambda_{4,00},\lambda_{4,01},\lambda_{4,02},\lambda_{4,11},\lambda_{4,12},\lambda_{4,22}). \end{array}$$

They fully determine the value of the equilibrium:

B 14 $$f_{i}^{\text{eq}}=\rho \exp\left[-(1+\lambda_{0}+\lambda_{1,\alpha }\xi_{i\alpha }+\lambda_{2,\alpha \beta }\xi_{i\alpha }\xi_{i\beta }+\lambda_{3,\alpha \beta \gamma }\xi_{i\alpha }\xi_{i\beta }\xi_{i\gamma }+\lambda_{4,\alpha \beta }\xi_{i\alpha }\xi_{i\beta }\xi_{i\kappa }\xi_{i\kappa })\right],$$

where the repeated sum of the second-, third- and fourth-order term involve only indices present in (B 13) (6 terms at second-order, 10 terms at third-order, 6 terms at fourth-order), for reasons of symmetry. The parameters *λ*_*k*_ must be determined to match the set of constraints of equation (2.12), expressed in this case by 26 constraints *G*_*k*_ = 0, *k* = 0…25, which are labelled with the same index system as the *λ*_*k*_ in equation (B 13). The first four constraints *G*_0_, *G*_1,*α*_ are the same as in the 5-moment model, equations (B 3) and (B 4), the following six constraints *G*_2,*αβ*_ are the same as in the 13-moment model, equation (B 8), the following ten constraints *G*_3,*αβγ*_ are the same as in the 20-moment model, and the remaining constraints are:

B 15 $$G_{4,\alpha \beta }=\sum_{i}\;f_{i}^{\text{eq}}\xi_{i\alpha }\xi_{i\beta }\xi_{i\kappa }\xi_{i\kappa }-2\rho [(E+2T)u_{\alpha }u_{\beta }+(E+T)T\delta_{\alpha \beta }]\;\text{(6 constraints)}.$$

The Jacobian of this model, which is easily derived using the same principles as the Jacobians of the earlier models in this appendix, is left out because of practical considerations. Eventually, 14- and 21-moment approaches can be built on top of their 13- and 20-moment counterparts by considering the trace of the constraint (B 15).

## Appendix C. Impact of shifted lattices on numerical equilibria-based LBMs

**(a) DVMs and adaptive LBMs**

In the framework of DVMs, discrete velocities are chosen according to the flow conditions in order to ensure the stability of the method. This is done by satisfying the following requirements in terms of flow velocity *u*_*α*_ [29]

C 1 $$\min_{i}(\xi_{i\alpha })\leq u_{\alpha }\leq \max_{i}(\xi_{i\alpha }),$$

and temperature *T*

C 2 $$\min_{i}|u_{\alpha }-\xi_{i\alpha }{|}^{2}\leq DT\leq \max_{i}|u_{\alpha }-\xi_{i\alpha }{|}^{2}.$$

Interestingly, the condition (C 1) translates to the fact that information cannot propagate at a speed below the smallest discrete velocity of the lattice, and above the maximal one—this condition is more commonly known as the (Courant, Friedrichs et Lewy) CFL condition. Similarly, the condition (C 2) translates to the fact that the temperature is bounded by the minimal and maximal internal energies of discrete velocities. Observing a lower limit for the temperature might be surprising at first, but in fact, this is exactly what is observed during simulations of both DVMs and LBMs (figures 1 and 2).

Consequently, by choosing the minimal and maximal norm of discrete velocities, and by centring the velocity discretization accordingly, researchers from the DVM community are able to obtain stable simulations that lead to accurate results even for very large velocity and temperature variations. In this context, the lattice can either be adjusted in a static [29] or dynamic [35] manner. Interestingly, both approaches share similarities with adaptive LBMs that were proposed by Sun *et al.* [102–104] in the late 1990s and early 2000s, and recently reinterpreted by Dorschner *et al.* [105]. Nevertheless, contrarily to the latter LBMs which suffer from conservation issues due to space interpolations [106], DVMs rely on finite-volume discretizations of the DVBE, hence preventing any conservation issue.

**(b) Integer value shifted lattices**

To avoid problems induced by the space interpolation, an alternative is to adjust the lattice to the flow by only considering discrete velocity components that have integer values. This is the main idea behind the concept of shifted lattices, as proposed by Frapolli *et al.* [107]. Interestingly, the latter methodology not only transposes the physical properties in the new reference frame (centred around the shift velocity *U*_*α*_, and the reference temperature *T*_0_), but also exactly transposes all the spectral properties (dissipation and dispersion) of the stream-and-collide algorithm in this reference frame [108,109].

Concretely speaking, the shifting approach requires the user to transpose the whole algorithm in the new reference frame. For the present approach, it means that only discrete velocities have to be adjusted through $\xi_{i\alpha }^{*}=\xi_{i\alpha }+U_{\alpha }$ without having to explicitly redefine the collision step, and more precisely the computation of the numerical equilibrium. Indeed, the Lagrange multipliers are computed through constraints that rely on the discrete velocities. Consequently, the computation of the numerical equilibrium self-adjust in an implicit manner to any change related to discrete velocities. This is not usually the case, and notably for analytical equilibria [108,110]. Assuming that the velocity bounds of the *unshifted* lattice are $u_{\alpha }^{\text{min}}$ and $u_{\alpha }^{\text{max}}$, then its *shifted* version will follow the condition (C 1), and eventually end up with the new velocity bounds

C 3 $$u_{\alpha }^{*,\text{min}}=u_{\alpha }^{\text{min}}+U_{\alpha }\;\text{and}\;u_{\alpha }^{*,\text{max}}=u_{\alpha }^{\text{max}}+U_{\alpha }.$$

Such behaviour is investigated hereafter for the Q39 and Q343 lattices, considering three sets of constraints (5, 10 and 13) as well as three shifting velocities along the *x*-axis, i.e. ***U*** = (*U*, 0, 0) with *U* ∈ {0, 1, 2}. The impact of the shift is quantified through error analysis related the macroscopic moment *M*_*xxαα*_, which allows us to compare these results with those presented in §3. Corresponding results are compiled in figure 12.

Qualitatively speaking, increasing *U* always widens the range of Ma numbers for which the Newton–Raphson solver does not encounter converge issues, and for *U* = 2, most configurations are stable for 0 ≤ Ma ≤ 4. Surprisingly, several optimal operating points (minimal macroscopic error) may appear when *U* increases. At first sight, this might be surprising, but after looking at results available in the LB literature, one can notice that similar results were also obtained for the entropic equilibrium derived through the MEP based on the conservation of mass and momentum only [108], as well as for LGCA [50].

Looking in more detail at results obtained for different combinations (shift and number of constraints), increasing the number of constraints seems to have a bigger impact on the convergence of the root-finding algorithm for higher values of *U*. In addition, while stability ranges are comparable for the 5- and 10-moment approaches, the equilibrium based on 13 constraints benefits less from the shift. Nevertheless, the error levels obtained with this approach remain very interesting.

All of this confirms the very promising behaviour of lower-order LBMs, relying on numerical equilibria based on *M* ≥ 10 constraints, for the simulation of supersonic flows with Ma > 2.
